# Mutations in *RNU7-1* Weaken Secondary RNA Structure, Induce MCP-1 and CXCL10 in CSF, and Result in Aicardi-Goutières Syndrome with Severe End-Organ Involvement

**DOI:** 10.1007/s10875-022-01209-5

**Published:** 2022-03-23

**Authors:** Leslie Naesens, Josephine Nemegeer, Filip Roelens, Lore Vallaeys, Marije Meuwissen, Katrien Janssens, Patrick Verloo, Benson Ogunjimi, Dimitri Hemelsoet, Program for Undiagnosed Rare Diseases (UD-PrOZA), Steven Callens, Bart Dermaut, Wim Terryn, Nika Schuermans, Bruce Poppe, Levi Hoste, Lisa Roels, Marieke De Bruyne, Elfride De Baere, Jo Van Dorpe, Amélie Dendooven, Anne Sieben, Gillian I. Rice, Tessa Kerre, Rudi Beyaert, Carolina Uggenti, Yanick J. Crow, Simon J. Tavernier, Jonathan Maelfait, Filomeen Haerynck

**Affiliations:** 1 Department of Internal Medicine and Pediatrics, Ghent University, 9000 Ghent, Belgium; 2 Primary Immunodeficiency Research Lab, Jeffrey Modell Diagnosis and Research Center, Ghent University Hospital, 9000 Ghent, Belgium; 3 VIB-UGent Center for Inflammation Research, 9052 Ghent, Belgium; 4 Department of Biomedical Molecular Biology, Ghent University, 9052 Ghent, Belgium; 5 Department of Pediatrics, Algemeen Ziekenhuis Delta, 8800 Roeselare, Belgium; 6 Department of Pediatrics, Algemeen Ziekenhuis Groeninge, 8500 Kortrijk, Belgium; 7 Department of Medical Genetics, University of Antwerp, 2000 Antwerp, Belgium; 8 Department of Medical Genetics, Antwerp University Hospital, 2650 Antwerp, Belgium; 9 Department of Pediatrics, Division of Pediatric Neurology, University Hospital Ghent, 9000 Ghent, Belgium; 10 Department of Pediatrics, Antwerp University Hospital, 2650 Edegem, Belgium; 11 Centre for Health Economics Research & Modeling Infectious Diseases (CHERMID), Vaccine & Infectious Disease Institute (VAXINFECTIO), University of Antwerp, 2610 Antwerp, Belgium; 12 Department of Neurology, Ghent University Hospital, 9000 Ghent, Belgium; 13 Center for Medical Genetics, Ghent University Hospital, 9000 Ghent, Belgium; 14 Department of Biomolecular Medicine, Ghent University, 9000 Ghent, Belgium; 15 Department of Pathology, Ghent University Hospital, 9000 Ghent, Belgium; 16 Department of Pathology, Antwerp University Hospital, 9000 Ghent, Belgium; 17 Division of Evolution, Infection and Genomics, School of Biological Sciences, Faculty of Biology, Medicine and Health, The University of Manchester, Manchester, UK; 18 Department of Hematology, Jeffrey Modell Diagnosis and Research Center, Ghent University Hospital, 9000 Ghent, Belgium; 19 VIB-UGent Center for Inflammation Research, Laboratory of Molecular Signal Transduction in Inflammation, VIB, 9052 Ghent, Belgium; 20 MRC Human Genetics Unit, Institute of Genetics and Cancer, The University of Edinburgh, Edinburgh, UK; 21 Laboratory of Neurogenetics and Neuroinflammation, University of Paris, Imagine Institute, Paris, France; 22 Department of Pediatric Pulmonology, Infectious Diseases and Immunology, Ghent University Hospital, Corneel Heymanslaan 10, Ghent, Belgium

**Keywords:** Aicardi-Goutières syndrome, AGS, Type I interferon, IFN-α, STAT phosphorylation, cGAS, U7 snRNP, Small nuclear RNA, RNU7-1

## Abstract

**Background:**

Aicardi-Goutières syndrome (AGS) is a type I interferonopathy usually characterized by early-onset neurologic regression. Biallelic mutations in *LSM11* and *RNU7-1*, components of the U7 small nuclear ribonucleoprotein (snRNP) complex, have been identified in a limited number of genetically unexplained AGS cases. Impairment of U7 snRNP function results in misprocessing of replication-dependent histone (RDH) pre-mRNA and disturbance of histone occupancy of nuclear DNA, ultimately driving cGAS-dependent type I interferon (IFN-I) release.

**Objective:**

We performed a clinical, genetic, and immunological workup of 3 unrelated patients with uncharacterized AGS.

**Methods:**

Whole exome sequencing (WES) and targeted Sanger sequencing of *RNU7-1* were performed. Primary fibroblasts were used for mechanistic studies. IFN-I signature and STAT1/2 phosphorylation were assessed in peripheral blood. Cytokines were profiled on serum and cerebrospinal fluid (CSF). Histopathology was examined on brain and kidney tissue.

**Results:**

Sequencing revealed compound heterozygous *RNU7-1* mutations, resulting in impaired RDH pre-mRNA processing. The 3′ stem-loop mutations reduced stability of the secondary U7 snRNA structure. A discrete IFN-I signature in peripheral blood was paralleled by MCP-1 (CCL2) and CXCL10 upregulation in CSF. Histopathological analysis of the kidney showed thrombotic microangiopathy. We observed dysregulated STAT phosphorylation upon cytokine stimulation. Clinical overview of all reported patients with *RNU7-1*-related disease revealed high mortality and high incidence of organ involvement compared to other AGS genotypes.

**Conclusions:**

Targeted *RNU7-1* sequencing is recommended in genetically unexplained AGS cases. CSF cytokine profiling represents an additional diagnostic tool to identify aberrant IFN-I signaling. Clinical follow-up of *RNU7-1*-mutated patients should include screening for severe end-organ involvement including liver disease and nephropathy.

**Supplementary Information:**

The online version contains supplementary material available at 10.1007/s10875-022-01209-5.

## Introduction

Type I interferons (IFN-Is), including IFN-α and IFN-β, are pivotal in the defense against viral infections [1]. However, chronic uncontrolled IFN-I production causes immunopathology and characterizes a group of autoinflammatory diseases referred to as type I interferonopathies. Aicardi-Goutières syndrome (AGS) is the prototypical type I interferonopathy and typically presents with neurologic regression during infancy [2–4]. Classic neuroradiologic AGS hallmarks include leukoencephalopathy, cerebral atrophy, and spot-like intracranial calcifications, resembling the sequelae of a congenital infection [5]. So far, seven single-gene inborn errors in nucleic acid metabolism or sensing are associated with the clinical phenotype of AGS: *TREX1*, *RNASEH2A*, *-B* and *-C*, *SAMHD1*, *ADAR*, and *IFIH1* [6]. Recently, biallelic mutations in *LSM11* and *RNU7-1* were reported in previously genetically uncharacterized cases of AGS. The Sm-like protein LSM11 and the U7 small nuclear (sn) RNA, which is encoded by the *RNU7-1* gene, are essential components of the U7 small nuclear ribonucleoprotein (snRNP) complex that mediates replication-dependent histone (RDH) pre-mRNA processing. Defects in RDH pre-mRNA processing result in accumulation of misprocessed polyadenylated histone transcripts and disruption of histone stoichiometry. It is hypothesized that the resulting exposure of nuclear DNA activates the innate immune receptor cGAS, driving production of IFN-Is [7]. To date, a total of 16 AGS cases in 11 independent pedigrees have been described with biallelic compound heterozygous mutations in *RNU7-1* [7]. Here, we report 3 additional unrelated AGS patients with compound heterozygous *RNU7-1* mutations, further substantiating the link between *RNU7-1* loss-of-function and AGS, and expanding the clinical, genetic, and immunological spectrum.

## Methods

### Whole Exome Sequencing and Sanger Sequencing

Whole exome sequencing (WES) of index and unaffected parents was performed on gDNA using SureSelect Human All Exon V5 kit (Agilent) or Twist Human Core Exome enrichment with additional probes for humane RefSeq transcripts (Twist Bioscience). Paired-end massively parallel sequencing (2 × 150 cycles) was performed on a NovaSeq 6000 (Illumina) (P1 and P2) or SureSelectXT Human All Exon V6 (Agilent) enrichment and HiSeq 3000 sequencing (Illumina) (P3). Data analysis was accomplished with an in-house developed pipeline, in accordance with international guidelines. For mutation detection in P1 and P2, VariantDB was used [8]. In addition, a genome-wide Human Phenotype Ontology (HPO)–based filtering was executed using the MOON software (Diploid). In P3, variants were annotated and filtered using in-house software. Bidirectional verification of the variants in *RNU7-1* was done by Sanger sequencing and subcloning of the PCR fragments used for sequencing with the Zero Blunt® TOPO® PCR Cloning Kit (Invitrogen; 450,245).

### Primary Cell Isolation and Culture

Primary human fibroblasts were isolated from a 5-mm skin punch biopsy. The epidermis was separated from dermis by scraping and afterwards cells were isolated by incubation at 37 °C with collagenase type II (Thermo Fisher Scientific) during at least 4 h on a spinning rotor. Primary fibroblasts were cultured in DMEM (Gibco) supplemented with 10% fetal bovine serum, penicillin 10,000 U/ml—streptomycin 10,000 U/ml (Gibco), L-Glutamine 200 mM (Gibco), 1 mM sodium pyruvate, and 2-Mercaptoethanol 50 mM (Gibco). The fibroblasts were maintained at 37 °C at 5% CO_2_. Cells were stored in 90% Fetal Calf Serum (FCS; Sigma-Aldrich; F7524) containing 10% dimethyl sulfoxide (DMSO) at − 150 °C until further use. Peripheral venous blood specimens were collected from age-matched healthy individuals and patients using EDTA and serum tubes. EDTA blood was diluted 1:2 in Hank’s Balanced Salt Solution (HBSS; Fisher Scientific; 24,020,117) and peripheral blood mononuclear cells (PBMCs) were isolated after gradient centrifugation over Ficoll-Paque (GE Healthcare; 17–1440-02). After two washes in cold HBSS, the yielded layer of PBMCs was counted in a Neubauer plate with trypan blue exclusion of dead cells. PBMCs were aliquoted in culture media and 10% dimethyl sulfoxide (DMSO; Sigma-Aldrich; D2650). Vials were placed in a − 80 °C freezer using controlled rate freezing in preparation for final storage at − 150 °C until further use. Serum tubes were spun at 4 °C and cell-free serum was subsequently aliquoted and stored at − 80 °C until analysis.

### Flow Cytometry Analysis of Phospho-STAT1/2 (p-STAT1/2) on PBMCs

Patient and age-matched healthy control PBMCs were thawed in 37 °C preheated complete RPMI medium; RPMI-1640 medium supplemented with GlutaMAX, 10% FCS, 1% penicillin–streptomycin (Pen/Strep; 10,000 U/mL; Gibco; 15,140,122), 1 mM sodium pyruvate (Gibco; 11,360,070), 1% non-essential amino acids (NEAA; Gibco; 11,140,035), and 50 μM 2- mercaptoethanol (Gibco; 31,350,010). In the setting of functional testing, cells were left to recover for 30 min at 37 °C and 5% CO_2_ after removal of DMSO. Cells were plated in a round bottom 96-well plate at a density of 0.5 × 10^6^ cells and washed with DPBS (Gibco) to remove culture medium. To allow analysis of PBMC subsets, cells were stained for 20 min at room temperature with monoclonal antibodies (mAbs) for extracellular epitopes, including CD4 (RPA-T4, BD), CD8 (RPA-T8, BD), CD19 (HIB19, Biolegend), CD14 (M5E2, BD), FcR blocking reagent (Miltenyi), and Fixable Viability Dye 506 (eBioscience) as live/dead marker in DPBS. After washing with serum-deprived culture medium, cells were stimulated for 15 min at 37 °C with IFN-α2 (1000 IU/mL), IFN-ω (1000 IU/mL), IFN-γ (50 ng/mL), IL-27 (100 ng/mL) or were left untreated. Cells were fixed in 4% paraformaldehyde (PFA, Sigma-Aldrich, #1,040,031,000) for 10 min at 37 °C. After washing with FACS buffer, the cells were permeabilized for 10 min in 100% methanol on ice. Cells were washed again and stained for 30 min at 4 °C with anti-STAT1-pY701 (14/P-STAT1, BD) and anti-STAT2-pY690 (D3P2P, Cell Signaling) mAbs in FACS buffer. Cells were washed with PBS and acquired with a BD LSRFortessa™. Data analysis was done with FlowJo software (v10.7.1).

### RNA Isolation and qRT-PCR Quantification

Primary fibroblasts (0.5 × 10^6^ cells) were lysed into RLT Plus-buffer (Qiagen) and stored at − 80 °C until further processing. RNA was obtained using the RNEasy Kit (QIAGEN) following the manufacturer’s instructions. Concentration and purity of RNA was assessed using the NanoDrop 2000 technology. One microgram of RNA was transcribed to cDNA using an oligo(dT)20 (ThermoFisher) primer and the SuperScript III First-Strand Synthesis System (ThermoFisher) for quantification of histone transcripts. Approximately 15 ng cDNA (estimated from input RNA) was used as input for quantitative Real-Time PCR (Lightcycler 480, Roche). Differences in cDNA input were corrected using normalization to SDHA cDNA levels. Relative quantitation of target cDNA was determined by the formula 2^–ΔΔCt^, with ΔΔCt denoting fold increases above the respective controls. SYBR Green primers (Integrated DNA Technologies) were used for qPCR of misprocessed histone RNA analysis. All SYBR Green primer sequences can be retrieved in Table S8.

### NanoString ISG Analysis

Blood was collected into PAXgene tubes (PreAnalytix) and, after being kept at room temperature for between 2 and 4 h, was frozen at − 80 °C until extraction. Total RNA was extracted from whole blood using a PAXgene (PreAnalytix) RNA isolation kit. Analysis of 24 genes and 3 housekeeping genes was conducted using the NanoString customer-designed CodeSets according to the manufacturer’s recommendations (NanoString Technologies). Data were processed with nSolver software (NanoString Technologies). The data were normalized relative to the internal positive and negative calibrators, the three reference probes, and the control samples. The median of the 24 probes for each of 27 HC samples was calculated. The mean NanoString score of the 27 HCs + 2 SD of the mean was calculated. Scores above this value (> 2.758) were designated as positive.

### Cytokine Quantification

Serum cytokines IL-1β, IL-1RA, IL-18, CXCL9, CXCL10, TNFα, and MCP-1 (CCL2) were quantified in duplicate by magnetic bead-based multiplex assay using xMAP technology (Luminex Corporation) and Bio-Plex assays, kits, and standards (Human Cytokine Screening Panel #12,007,919; Bio-Rad, and Human Inflammation Panel #171DL0001; Bio-Rad) according to the manufacturer’s protocol. Multiplex assay samples were diluted at 1:2. Acquisition and analysis was performed on a Bio-Plex 200 reader and using the Bio-Plex Manager software (Bio-Rad). The S-PLEX human IFN-α2a kit by MSD (Meso Scale Discovery) was used for determining IFN-α2 in serum samples following the manufacturer’s instructions.

### Statistics

Data values were analyzed using the non-parametric unpaired Mann–Whitney *U* test or Student’s *t*-test (when indicated). Significance levels were denoted as < 0.05 (*), < 0.01 (**), < 0.001 (***), < 0.0001 (****). Statistical analyses were performed with Prism v9 (GraphPad software).

## Results

### Compound Heterozygous *RNU7-1* Mutations in 3 Patients with an AGS Phenotype

**Fig. 1:**
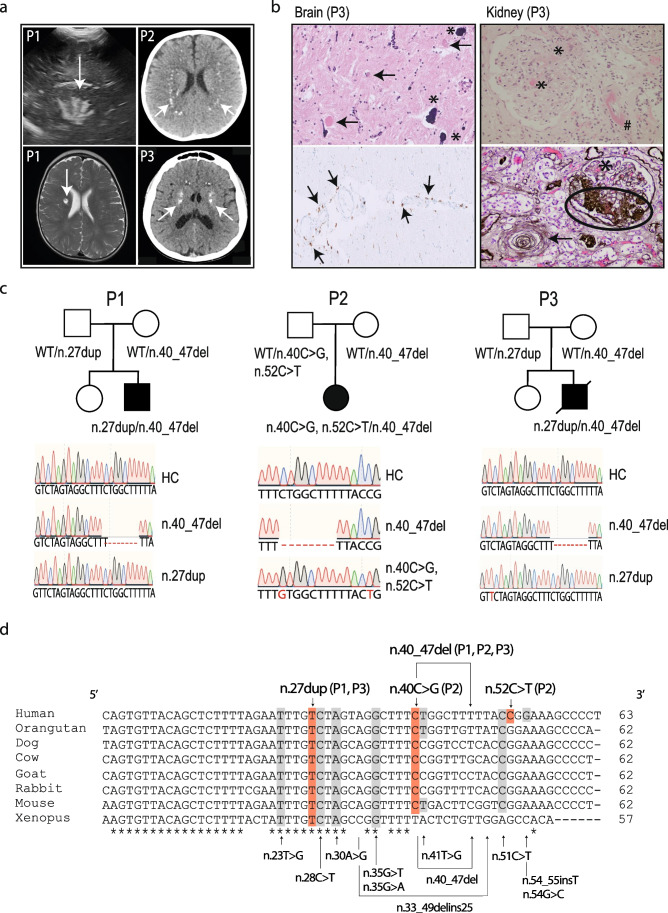
AGS patients harboring compound heterozygous *RNU7-1* mutations. **a** Cranial ultrasound of P1 revealed hyperechogenic lines located in the area of lenticulostriate vessels, a radiological image compatible with lenticulostriate vasculopathy (left upper panel). Lenticulostriate ischemic lacunar cerebral infarct on T2-weighted MRI image of P1 (left lower panel). Neuroimaging of P2 and P3 showing spot-like calcifications periventricular and in the basal ganglia on brain CT (right upper and lower panel). **b** Postmortem histologic examination of brain tissue of P3 revealed numerous calcifications in the walls of arterioles and capillaries (*) with granulovacuolar degeneration of neurons (arrow) adjacent to the affected vessels (left upper panel, 20 ×). Centrum semi-ovale exhibited perivascular crowding of CD68 + activated microglia and histiocytes (left lower panel, 20 ×). Hematoxylin and eosin (HE) stain of the kidney showed a glomerulus with fibrin thrombi (*) and an arteriole with intramural fibrin precipitation (#) (right upper panel, 200 ×). Silver staining (Jones methenamine) of a glomerulus with mesangiolysis and endothelial swelling (*), segmental sclerosis (oval), and a nearby arteriole with “onion-skinning” (arrow) (right lower panel, 200 ×). **c** Familial pedigree of patients harboring biallelic mutations in *RNU7-1* with electropherograms in comparison to a healthy control (HC). **d** Clustal Omega alignment of *RNU7-1* homologs with mutations of P1, P2, and P3 depicted above (red) and pathogenic variants in *RNU7-1* reported to data below (gray) [7]

We investigated 3 patients with AGS from unrelated non-consanguineous families of European origin (full clinical descriptions are given in supplemental data S1). All 3 patients presented with classical features of AGS including psychomotor retardation, spastic quadriparesis, and intracranial calcifications (Fig. 1a). In addition to calcifications, ultrasound imaging revealed lenticulostriatal vasculopathy in P1 (Fig. 1a). P1 and P2 are in clinical follow-up and currently 2 and 3 years old, respectively. At 19 years of age, P3 developed tonic–clonic convulsions. Laboratory analysis of P3 revealed mild hemolytic anemia and severe renal insufficiency (creatinine of 2.85 mg/dl, eGFR 30 ml/min) with arterial hypertension. Following palliative care, he died 1 month later. Histological examination of the brain demonstrated calcifications in the walls of arterioles and capillaries, demyelination of neurons, and gliosis (Fig. 1b). Perivascular clustering of histiocytes and CD68 + microglial cells could be observed (Fig. 1b). Histopathology of the kidney showed fibrin thrombi and intimal proliferation, hallmarks of thrombotic microangiopathy (Fig. 1b).

WES in these 3 patients did not reveal pathogenic variants in the seven well-known AGS-related genes nor in the recently described *LSM11* gene (see Table S2 for 20 × coverage). Subsequently, we performed Sanger sequencing and found biallelic mutations in *RNU7-1* (NR_023317.1), encoding U7 snRNA, in these 3 patients. All patients (P1, P2, and P3) carried an 8 bp deletion in *RNU7-1* (n.40_47del), which has been recently associated with AGS [7]. In addition, we identified a novel mutation in *RNU7-1* absent in the Genome Aggregation Database (gnomAD); P1 and P3 carried a n.27dup mutation in trans. In P2, we detected a n.40C > G mutation in trans, which has been reported only once in Gen egnomAD. In conclusion, Sanger sequencing revealed that P1, P2, and P3 (postmortem analysis) were carrying compound heterozygous mutations in *RNU7-1* (Fig. 1c, d).

### *RNU7-1* Mutations in the 3′ Stem-Loop Decrease Stability of Secondary RNA Structure

**Fig. 2:**
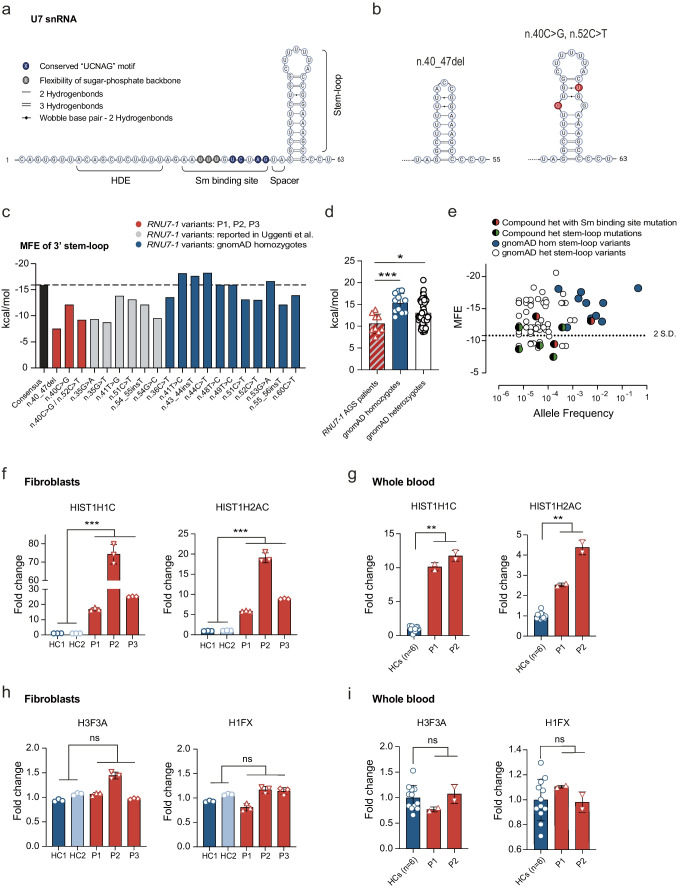
*RNU7-1* mutations in the 3′ stem-loop weaken secondary structure and result in RDH pre-mRNA misprocessing. **a** Secondary structure and functional domains of U7 snRNA. Correct assembly of a functional U7 snRNP requires the flexibility of the sugar-phosphate backbone by uridines 23–25 (gray), a conserved “UCNAG” motif (blue) within the Sm binding site, spacing of two or more nucleotides between stem-loop and the Sm binding site, and a stable 3′ stem-loop. **b** Secondary structure of n.40_47del and n.40C > G/n.52C > T stem-loop mutations. **c** MFE of 3′ stem-loop consensus sequence (black), *RNU7-1* variants of P1, P2, P3 (red), reported *RNU7-1* mutations in AGS patients (gray), and homozygous carriers in gnomAD (blue). **d** MFE comparison of biallelic *RNU7-1* mutations found in AGS patients compared to gnomAD homozygote and heterozygote *RNU7-1* variants. Statistical analysis using the non-parametric unpaired Mann–Whitney *U* test (*** *P* < 0.001, * *P* < 0.5). Mean with s.d. is shown. **e** Graph of MFE against allele frequency for *RNU7-1* stem-loop mutations in AGS patients, heterozygous and homozygous stem-loop variants in gnomAD. **f**, **g** Oligo(dT)-primed reverse transcription of polyadenylated RNA including replication-dependent linker (HIST1H1C) and core (HIST1H2AC) histones in primary fibroblasts (**f**) or whole blood (**g**) of *RNU7-1* patients and HCs. **h**, **i** Expression of polyadenylated replication-independent histones (H3F3A, H1FX) in primary fibroblasts (**h**) or whole blood (**i**) of *RNU7-1* patients and HCs. Data (**f**–**i**) presented as fold change normalized to cellular *SDHA* relative to the values for HCs. Data shown are representative for two (**f**–**i**) independent experiments with mean and s.d. of ***n*** = 3 technical replicates (f, h) or ***n*** = 2 technical replicates (**g**, **i**). **P < 0.01, ***P < 0.001, (**f**–**i**) by non-parametric unpaired Mann–Whitney *U* test

The n.27dup mutation (P1, P3) is located in the Sm binding site, the n.40C > G mutation (P2) and the n.40_47del mutation (P1, P2, P3) in the 3′ stem-loop of U7 snRNA (Fig. 2a, b) [7]. The Sm binding site is crucial for the correct assembly of the U7 snRNP [9]. Furthermore, mutations at positions n.23, n.28, and n.30, all located within the Sm binding site, were identified as pathogenic [7]. As such, the patient-derived n.27dup mutation was predicted to impair U7 snRNP assembly. The assessment of pathogenicity of variants in non-protein encoding genes, such as *RNU7-1*, is challenging. It is not possible to infer damaging status (such as by recognizing a variant as null) and commonly used in silico algorithms are unavailable. To evaluate the structural impact of the 3′ stem-loop mutations of our patients, we performed secondary structure analysis using the RNAfold web server and calculated the minimum free energy (MFE) in which a more negative free energy value represents a more stable structure [10]. All predicted secondary structures containing the previously reported and the newly identified 3′ stem-loop mutations had a more positive MFE and thus reduced stability compared to the consensus sequence (Fig. 2c, Fig. S3). In P2, the *RNU7-1* allele containing the n.40C > G mutation also harbored a n.52C > T polymorphism, which was predicted to further destabilize the 3′ stem-loop structure in an additive manner (Fig. 2b, c). The average MFE of homozygous 3′ stem-loop variants in *RNU7-1* reported in gnomAD was significantly more negative compared to the average MFE in patients harboring *RNU7-1* mutations, suggesting a more stable secondary structure (Fig. 2c, d). In line with the autosomal recessive nature of *RNU7-1*-mediated AGS, the heterozygous *RNU7-1* variants reported in gnomAD spanned the whole spectrum, containing both neutral variants and destabilizing mutations (Fig. 2d, e). Severely destabilizing heterozygous variants (defined as < 2SD of average MFE of homozygous variants or > − 10.81) were rare (Fig. 2e) with a number of mutant alleles (MFE > − 10.81) in the population database of 205 out of 152,312 sequenced alleles (gnomAD v3.1) or an estimated 0.135% mean allele frequency (MAF). Some AGS patients harbored *RNU7-1* stem-loop mutations with a MFE < − 10.81 (Fig. 2e). Notably, these less destabilizing mutations were always compound heterozygous with mutations in the Sm binding site or stem-loop mutations with a MFE > − 10.81 (Fig. 2e).

### *RNU7-1* Mutations in the 3′ Stem-Loop or Sm Binding Site Result in RDH Pre-mRNA Misprocessing

Next, we set out to assess the functional consequences of the identified *RNU7-1* mutations. The U7-snRNP complex is involved in RDH pre-mRNA processing. Impaired assembly of U7 snRNP disrupts endonucleolytic cleavage of the 3′ end of RDH pre-mRNAs, preserving the poly(A) tail. We performed RT-qPCR using oligo(dT) primers which anneal to the poly(A) sequences of aberrant RDH mRNA isoforms. In line with previous findings [7], we observed increased misprocessed polyadenylated mRNAs of both linker (HIST1H1C) and core (HIST1H2AC) RDH in patient fibroblasts (P1, P2, P3) as well as in whole blood (P1, P2) (Fig. 2f, g). Normal protein expression of LSM11, an essential component of the U7-snRNP, was confirmed in primary fibroblasts (Fig. S4). Expression of replication-independent histone mRNAs, which are normally polyadenylated and do not require the U7 snRNP complex, was unaffected (Fig. 2h, i).

### An IFN-I Signature in Peripheral Blood of Patients with Biallelic *RNU7-1* Mutations

**Fig. 3:**
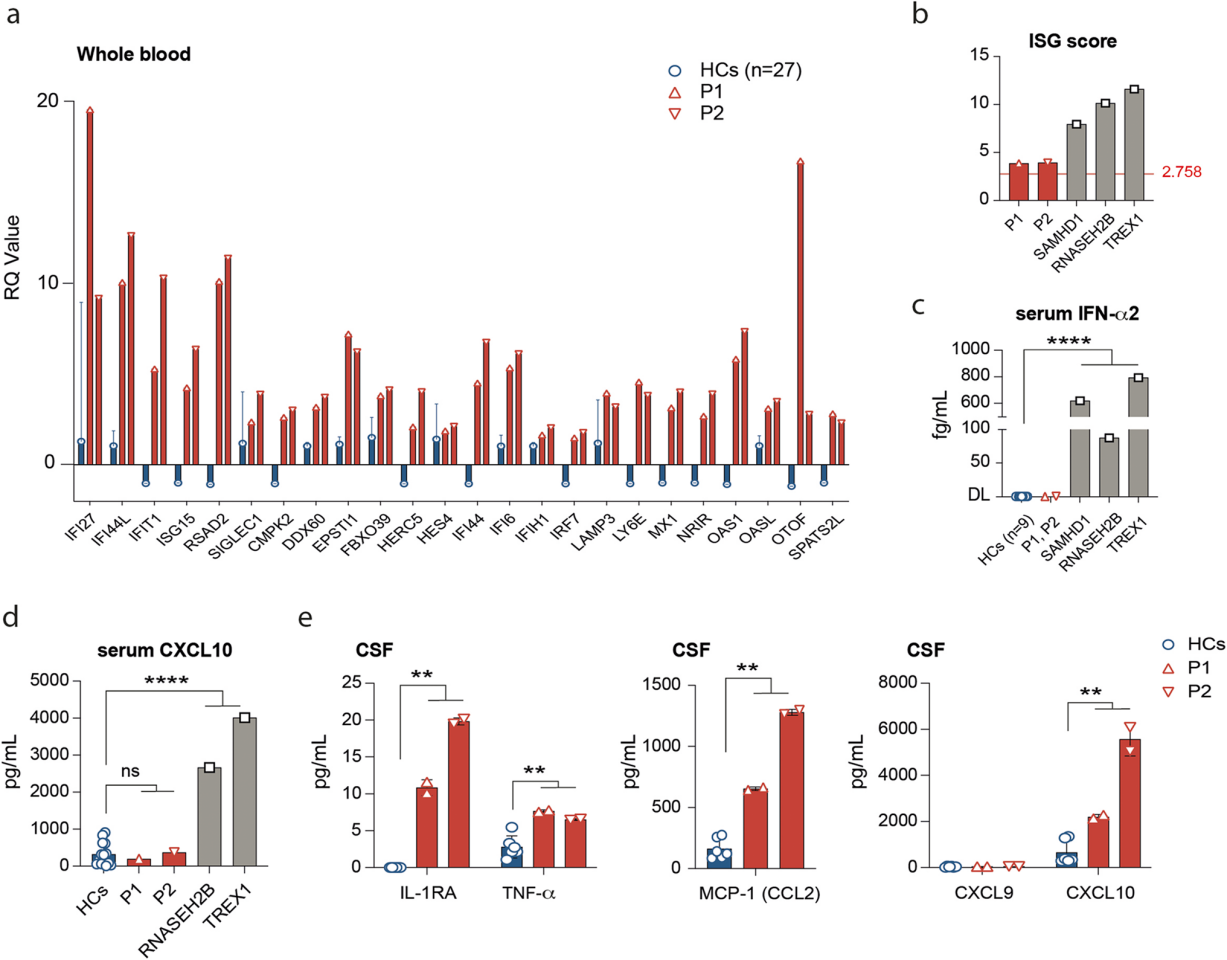
AGS patients P1 and P2 display a systemic type I interferon signature and increased IFN-I-inducible cytokines in CSF. **a** Relative quantification (RQ) of 24 ISGs in whole blood of *RNU7*-*1*-mutated patients (P1, P2) compared to healthy controls (*n* = 27). **b** ISG scores calculated from the median fold induction of a set of 24 ISGs (see “Methods,” normal < 2.758) of P1, P2, and AGS patients without *RNU7-1* mutations, but harboring biallelic mutations in *SAMHD1* (*n* = 1), *RNASEH2B* (*n* = 1), or *TREX1* (*n* = 1). **c** Measurement of IFN-α2 in serum by MSD (Meso Scale Discovery) in P1 and P2 compared to HCs (*n* = 9) and other AGS genotypes (DL, detection limit). **d** Measurement of CXCL10 in the serum of P1 and P2 compared to HCs (*n* = 14) and other AGS genotypes. **e** Cytokine profiling in CSF including IL-1RA, TNF, MCP-1 (CCL2), CXCL9, and CXCL10 of P1 and P2 compared to HCs (*n* = 3). Data shown represents mean and s.d. of *n* = 2 technical replicates. **P < 0.01, ****P < 0.0001, by non-parametric unpaired Mann–Whitney *U* test

We examined an IFN-I signature by quantifying the mRNA expression of 24 interferon-stimulated genes (ISGs) measured on a NanoString platform in whole blood of our AGS patients compared to HCs (Fig. 3a, see “Methods”). Both P1 and P2 demonstrated a mildly elevated ISG score (respectively 3.86 and 3.94) in contrast with a control group of three AGS patients with biallelic mutations in *SAMHD1* (7.94), *RNASEH2B* (10.14), or *TREX1* (11.60) (Fig. 3b, Table S5, case descriptions in supplemental data S1). In line with the pronounced IFN-I signature in *SAMHD1*, *RNASEH2B*, and *TREX1* patients, increased protein concentrations of IFN-a2 could be readily identified in the serum of these patients, but not in our *RNU7-1*-mutated AGS patients (Fig. 3c).

### Increased Concentrations of CXCL10 and MCP-1 Cerebrospinal Fluid of *RNU7-1* Patients

We quantified IFN-I-induced cytokines in serum of AGS patients. Correlating with the high ISG score and serum IFN-α2 levels, we observed pronounced CXCL10 levels in the serum of patients harboring *RNASEH2B* (2663 pg/mL) and *TREX1* (4010 pg/mL) mutations, whereas the concentration in both *RNU7-1*-mutated patients (P1: 367 pg/mL, P2: 194 pg/mL) was comparable to HCs (median 232 pg/mL, IQR 66–546) (Fig. 3d). In parallel, we performed cytokine profiling on cerebrospinal fluid (CSF) of *RNU7-1* patients (P1, P2). IL-1b, IL-6, and IL-18 could not be detected (data not shown). Low concentrations of IL-1RA (P1: 10 pg/mL, P2: 19 pg/mL), produced by monocytes upon IFN-I stimulation [11], were present in the CSF of patients with *RNU7-1* mutations but undetectable in HCs (Fig. 3e). Evaluation of the IFN-I-inducible cytokines monocyte chemoattractant protein 1 (MCP-1 or CCL2) (P1: 644 pg/mL, P2: 1263 pg/mL) and CXCL10 (P1: 2274 pg/mL, P2: 6089 pg/mL) revealed a remarkable increase in the CSF compartment (Fig. 3e). The IFN-g-responsive cytokine CXCL9 could not be detected (Fig. 3e).

### STAT1/2-Dependent Cytokines Display Aberrant Signaling in Monocytes of Patients with AGS

**Fig. 4:**
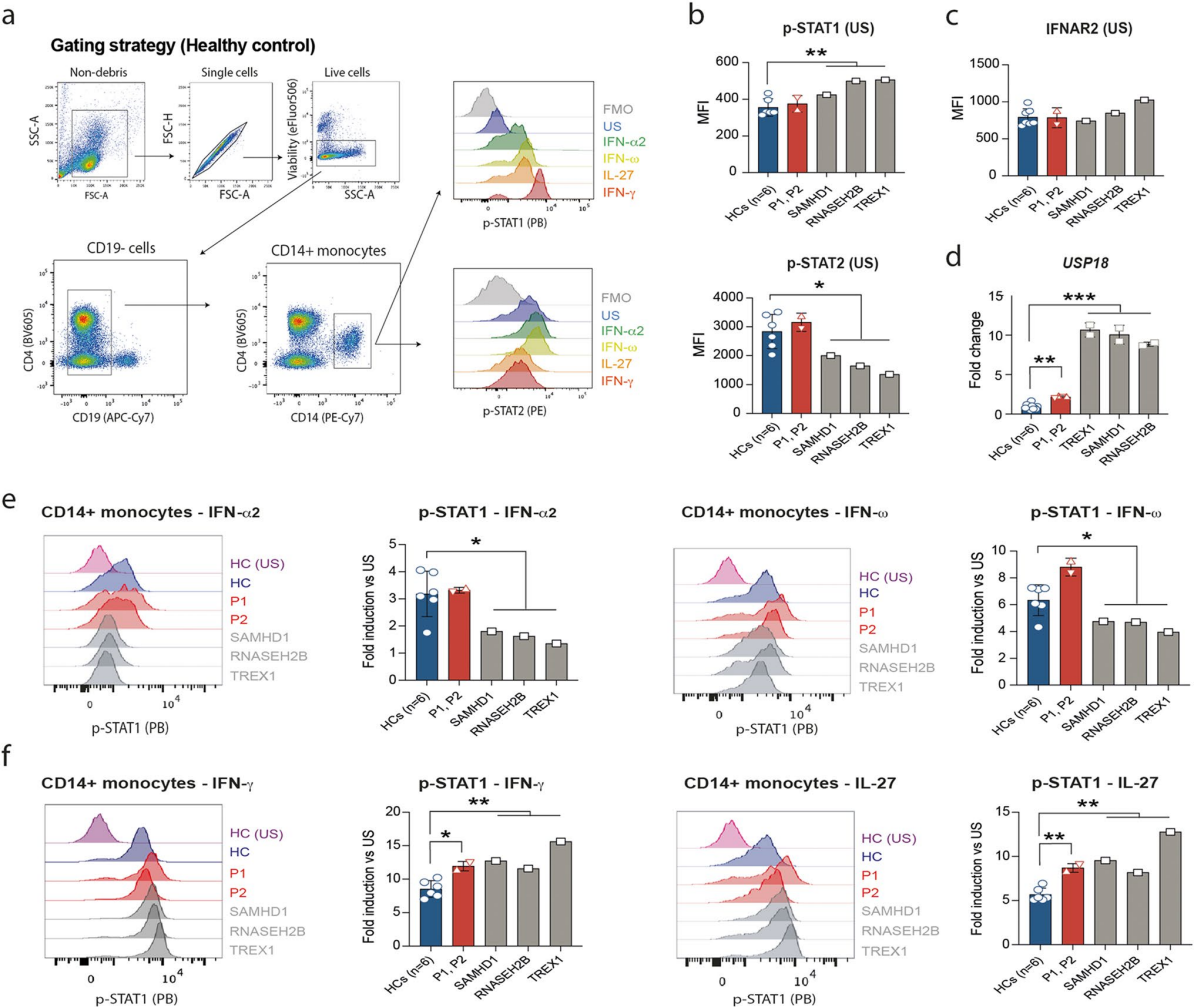
Dysregulated STAT1/2 signaling in monocytes of patients with AGS upon stimulation with IFN-Is and STAT2 independent cytokines. **a** Flow cytometry gating strategy on healthy control sample used to identify CD14 + monocytes and mean fluorescence intensity (MFI) of p-STAT1 (Y701) and p-STAT2 (Y690). **b** Unstimulated (US) phosphorylation levels of STAT1 (pY701) and STAT2 (pY690) in monocytes. **c** Extracellular staining of IFNAR2 (interferon-α/β receptor 2) on unstimulated monocytes. **d** mRNA expression of *USP18* in whole blood of *RNU7-1*-mutated patients and AGS patients harboring biallelic mutations in *SAMHD1* (*n* = 1), *RNASEH2B* (*n* = 1), or *TREX1* (*n* = 1) compared to HCs (*n* = 6), presented as fold change normalized to cellular *SDHA* relative to the values for HCs. Data shown (**d**) are representative for two independent experiments (mean and s.d. of *n* = 2 technical replicates). **P < 0.01, ***P < 0.001, by non-parametric unpaired Mann–Whitney *U* test. **e** Phosphorylation of STAT1 (pY701) in CD14 + monocytes after stimulation with STAT2 dependent IFN-α2 and IFN-ω cytokines, presented as fold change normalized to the basal respective phosphorylation. **f** Phosphorylation of STAT1 (pY701) in CD14 + monocytes after stimulation with STAT2 independent IFN-γ and IL-27 cytokines, presented as fold change normalized to the basal respective phosphorylation. Data shown are representative for two (**b**, **c**, **e**, **f**) independent experiments with each point representing one biological replicate. *P < 0.05, **P < 0.01, (**b**, **c**, **e**, **f**) by unpaired Student’s *t*-test

Finally, we interrogated STAT1/2 phosphorylation in CD14^+^ monocytes upon ex vivo stimulation of peripheral blood mononuclear cells (PBMCs) with IFN-α2, IFN-ω, IL-27, and IFN-γ (Fig. 4a). PBMCs of patients with AGS harboring mutations in *SAMHD1*, *RNASEH2B*, or *TREX1* were used as controls. Similar to chronic IFN-I stimulation in vitro [12], tyrosine phosphorylation of residue Y690 on STAT2 (but not pY701-STAT1) was decreased in unstimulated CD14^+^ monocytes of AGS patients with mutations in *SAMHD1*, *RNASEH2B*, and *TREX1* (Fig. 4b)*.* Decreased STAT2 phosphorylation was not due to impaired IFNAR (interferon-α/β receptor) expression (Fig. 4c). Quantifying mRNA expression of negative regulators of JAK-STAT signaling such as *SOCS1*, *SOCS3*, and *USP18*, we observed significantly increased expression levels of *USP18*, which correlated with the ISG score (Fig. 4d). Of note, mRNA expression of *STAT1* was normal and *STAT2* mRNA expression mildly upregulated compared to HCs (Fig. S6). Upon stimulation with IFN-α2 and IFN-ω (IFN-Is; STAT2 dependent), the AGS genotypes (*SAMHD1*, *RNASEH2B*, *TREX1*) with a prominent IFN-I signature in peripheral blood exhibited impaired Y701 phosphorylation of STAT1 (Fig. 4e). In contrast, stimulation with IFN-γ and IL-27 (both STAT2 independent) resulted in increased pY701-STAT1 in CD14^+^ monocytes of patients with mutations in *RNU7-1* and other AGS genotypes (Fig. 4f).

### Clinical Overview of Patients with *RNU7-1* Mutations Reveals High Mortality and End-Organ Involvement

Classical AGS presents in the first few months after birth with psychomotor retardation and neurological features including epileptic seizures, motor disorders (dystonia/spasticity), eye movement abnormalities, or spastic paraparesis [3]. To gain further insight into the clinical phenotype associated with mutations in *RNU7-1*, we incorporated the clinical findings of our 3 cases in the previously described cohort of patients with *RNU7-1*-related disease (Table 1) [7]. Psychomotor retardation as well as spasticity and dystonia is present in all patients (19/19, 100%). Neuroradiologic imaging revealed intracranial calcifications (16/19, 84%) and leukodystrophy (14/19, 74%) in most patients. In addition, a clinically relevant association with liver damage (10/19, 53%), arterial hypertension (7/19, 37%), pericardial effusions (5/19, 26%), and kidney disease (8/19, 42%), as observed in P3, is found in *RNU7-1* patients. Moreover, *RNU7-1* patients have a median age of 9 years and the highest mortality rate (8/19, 42%) among AGS genotypes (Fig. S7).

**Table 1:** Overview of demographic features and clinical manifestations of patients with compound heterozygous *RNU7-1* mutations. *M*, male; *F*, female; *A*, alive; *D*, deceased; *U*, unknown; + , present; -, absent

	P1	P2	P3	Other *RNU7-1* patients (*n* = 16) [7]
Demographics				
Current age (y)	2	3	19	9 (2–23)
Sex	M	F	M	9 M / 7 F
Survival	A	A	D	9 A / 7 D
Neurological features (19/19, 100%)				
Irritability, feeding difficulties, failure to thrive	+	-	+	7/16 (44%)
Psychomotor retardation	+	+	+	16/16 (100%)
Spasticity/hypertonia	+	+	+	16/16 (100%)
Microcephaly	-	-	+	3/16 (19%)
Epilepsy	+	-	+	3/16 (19%)
Sensory neuropathy	-	-	-	4/16 (25%)
Nystagmus	-	-	-	1/16 (6%)
Neuroimaging (19/19, 100%)				
Leukodystrophy	-	+	+	12/16 (75%)
Intracranial calcifications	+	+	+	13/16 (81%)
Cerebral atrophy	+	+	+	7/16 (44%)
Lenticulostriate vasculopathy	+	-	-	0/16 (0%)
Ischemic stroke	+	-	-	0/16 (0%)
Abnormal myelination	+	+	+	4/16 (25%)
Cystic lesions	-	-	-	1/16 (6%)
Kidney disease (8/19, 42%)				
Renal insufficiency	-	-	+	5/16 (31%)
Proteinuria	-	-	+	2/16 (13%)
Thrombotic microangiopathy	-	-	+	1/16 (6%)
Echogenic kidneys	U	U	-	1/16 (6%)
Glomerulosclerosis	U	U	+	1/16 (6%)
Cysts	U	U	+	1/16 (6%)
Hematuria	-	-	-	1/16 (6%)
Liver disease (10/19, 53%)				
Transaminitis	-	+	-	8/16 (50%)
Hepatomegaly	-	-	-	2/16 (13%)
Edema and hypoalbuminemia	-	-	-	2/16 (13%)
Steatosis/fibrosis	U	U	-	2/16 (13%)
Skin manifestations (9/19, 47%)				
Livedo reticularis	-	-	-	2/16 (13%)
Acrocyanosis	-	-	-	2/16 (13%)
Plantar erythema	-	-	+	0/16 (0%)
Dry skin	+	-	-	2/16 (13%)
Chilblains	-	-	-	2/16 (13%)
Poikiloderma	-	-	-	1/16 (6%)
Other				
Arterial hypertension	-	-	+	6/16 (38%)
Optic atrophy	-	-	U	2/16 (13%)
Hypothyroidism	-	-	-	5/16 (31%)
Pericardial effusion	U	U	+	4/16 (25%)
Anemia	-	-	+	4/16 (25%)
Recurrent urinary tract infections	-	-	+	2/16 (13%)
Pancreatitis	-	-	-	1/16 (6%)
Osteoporosis with fractures	-	-	-	1/16 (6%)
Cryptorchidism	-	-	+	0/16 (0%)
Micropenis	-	-	-	1/16 (6%)
Scoliosis	-	-	-	1/16 (6%)
Hypospadias	+	-	-	0/16 (0%)
Chronic conjunctivitis	-	-	+	0/16 (0%)
Synovitis	-	-	+	0/16 (0%)

## Discussion

We performed in-depth analysis of 3 unrelated patients presenting with a genetically unexplained AGS and found compound heterozygous *RNU7-1* mutations underlying disease. *RNU7-1* starts 206 base pairs upstream of the first exon of the protein-coding *C12orf57* gene (NM_138425.4). Since *RNU7-1* is a noncoding snRNA gene, WES coverage is suboptimal given that commercially available exome capture kits predominantly target protein-coding gene regions. Indeed, in this study, we made use of Sanger sequencing to identify the mutations in *RNU7-1*. We propose that the diagnostic workup for genetically unexplained AGS cases should include targeted *RNU7-1* sequencing or whole-genome sequencing (WGS).

U7 snRNA is transcribed by RNA polymerase II and comprises three distinct regions. The 5′ end is complementary to the 3′ UTR of RDH pre-mRNA (Histone Downstream Element; HDE site) and is followed by a noncanonical Sm binding site and a 3′ stem-loop [13]. Mutagenesis experiments revealed critical regions within the U7 snRNA Sm binding site that determine the correct assembly of the U7 snRNP [9]. Furthermore, a previous study identified mutations at positions n.23, n.28, and n.30, all located within the Sm binding site as pathogenic [7]. As such, the patient-derived n.27dup mutation is predicted to impair U7 snRNP assembly. The formation of functional U7 snRNP also requires a stable 3′ stem-loop [9]. To evaluate the structural impact of 3′ stem-loop mutations of our, and other reported, *RNU7-1*-mutations, including the novel n.40C > G mutation in P2, we made use of the RNAfold web server to predict secondary structure formation [10]. The folding stability of U7 snRNA can be assessed by calculating the MFE, in which a lower MFE is representative of a more stable structure. *RNU7-1* mutations of AGS patients in the 3′ stem-loop have a reduced stability of the secondary U7 snRNA structure as calculated by the MFE. We also calculated the MFE of homozygous variants in *RNU7-1* reported in gnomAD, containing WGS data of presumably healthy individuals [14]. Of interest, all homozygous variants in *RNU7-1* reported in gnomAD are located in the 3′ stem-loop. Our results showed that these homozygous variants have no or modest impact on folding stability, further confirming the validity of our findings. Finally, the *RNU7-1* mutations in our patients with AGS disrupted RDH pre-mRNA processing shown by accumulation of misprocessed polyadenylated mRNAs. This selective aberrant RDH pre-mRNA processing was in line with the in silico predicted pathogenicity based on MFE and confirmed that the *RNU7-1* mutations underlying AGS in our patients result in defective U7 snRNP function.

Uncontrolled IFN-I production is a pathognomonic feature of AGS. Thethering of the nuclear-localized DNA sensor cGAS to histone 2A-histon 2B nucleosomes is crucial to prevent genomic DNA recognition [15, 16]. The disturbed histone stoichiometry in *RNU7-1* patients might release cGAS from the nucleosomes, facilitating DNA recognition and cGAS activation. Indeed, confocal imaging of fibroblasts derived from patients with compound heterozygote *RNU7-1* mutations revealed DNA-induced liquid phase condensation of activated cGAS molecules in the nucleus [7, 17]. This autoreactivity to self-DNA then triggers the production of the second messenger molecule 2′3′-cyclic GMP-AMP (cGAMP) causing chronic IFN-I production via the activation of STING [18]. Our immunological studies revealed a discreetly elevated ISG signature in peripheral blood in patients with compound heterozygous *RNU7-1* mutations. However, the data in Uggenti et al. indicate that in the vast majority of patients with compound heterozygote *RNU7-1* mutations the ISG signature was similar to other AGS-related genotypes [7]. ISG signatures in patients with AGS are known to vary over time, rendering early AGS diagnosis challenging. We performed cytokine profiling in serum and CSF and found a remarkable increase of the IFN-I-inducible cytokines MCP-1 (CCL2) and CXCL10 in the CSF compartment of *RNU7-1* patients. This discrepancy of cytokine profiling in peripheral blood versus CSF in AGS suggests a distinct anatomical compartmentalization of the disease process [19], and highlights the diagnostic utility of these markers in the CSF of AGS patients as suggested in a previous report [20].

Finally, we interrogated STAT1/2 phosphorylation and found unexpected, pleiotropic effects on cytokine signaling in AGS patients. STAT2 is known for its involvement in IFN-I/III signaling pathways and is essential in antiviral immunity. In recent years, STAT2 has also been shown to participate in negative regulatory activity towards cytokine signaling pathways [21, 22]. For instance, STAT2-dependent USP18 recruitment to the type I IFN receptor subunit IFNAR2 is required for USP18-mediated receptor dimerization interference [21, 23, 24]. The increased upregulation of *USP18* in patients with AGS might explain the observed impairment of STAT1 phosphorylation upon stimulation with IFN-I. In addition, unphosphorylated STAT2 is constitutively bound to STAT1 via a conserved interface [23]. Upon stimulation with STAT1-dependent cytokines, this interaction inhibits phosphorylated STAT1 to form homodimers and translocate to the nucleus [23]. This regulatory function of STAT2 could be abrogated upon recruitment to USP18 and might explain the increased pY701-STAT1 in our patients with AGS upon stimulation with STAT1-dependent cytokines (e.g., IFN-γ and IL-27). Our results indicate that much remains to be learned about the regulatory functions of STAT2 in steady state and in the context of chronic IFN-I exposure. Furthermore, we speculate that the dysregulated JAK-STAT signaling might underlie some of the unexplained clinical characteristics in patients with AGS.

Clinical overview of patients with *RNU7-1*-related disease revealed high mortality and high incidence of organ involvement such as liver and kidney disease, including thrombotic microangiopathy. Moreover, the high mortality in *RNU7-1* patients was mostly attributed to these additional organ-specific co-morbidities (Fig. S7). Of importance, these pathologies have not been reported to such extent in other AGS genotypes [25]. Several hypotheses remain plausible. On the one hand, U7 snRNP dysfunction affects the maturation of replication-dependent histones which are required during cell proliferation for the packaging of newly synthetized DNA [26]. Our studies revealed more pronounced accumulation of misprocessed polyadenylated histone transcripts in actively proliferating fibroblasts compared to whole blood in our patients. As such, tissue-specific disrupted histone stoichiometry might drive the particular phenotype underlying *RNU7-1*-driven pathology. On the other hand, *RNU7-1* and *LSM11* are the first AGS genes that do not encode for proteins with a known role in nucleoside sensing or metabolism. The specific end-organ manifestations in patients with compound heterozygous mutations in *RNU7-1* might also be the consequence of a disturbed nucleosome structure, an essential housekeeping protein complex.

In conclusion, biallelic mutations in *RNU7-1* expand the genetic etiology of AGS. The in-depth genetic, immunological, and clinical evaluation presented in this study reveals the requirement of targeted sequencing of *RNU7-1* (or implementation of WGS) and hint to the complex immunopathology to be considered in the diagnostic workup and research for potential AGS treatments.

## Supplementary Information

Below is the link to the electronic supplementary material.

Supplementary file1 (JPG 462 KB)

## Data Availability

Anonymized data will be shared by request from any qualified investigator.
