# Supplementary material for: Mutations in RNU7-1 Weaken Secondary RNA Structure, Induce MCP-1 and CXCL10 in CSF, and Result in Aicardi-Goutières Syndrome with Severe End-Organ Involvement
Source: J Clin Immunol. 2022 Mar 23;42(5):962–74. doi: 10.1007/s10875-022-01209-5 (PMC9402729; doi:10.1007/s10875-022-01209-5)
Supplement: Supplementary file 1 — Supplementary file1 (JPG 462 KB) [file 10875_2022_1209_MOESM1_ESM.jpg]

**Mutations in *RNU7-1* weaken secondary RNA structure, induce MCP-1 and CXCL10 in CSF and result in Aicardi-Goutières syndrome with severe end-organ involvement**

**Leslie Naesens^1,2^, Josephine Nemegeer^3,4^, Filip Roelens^5^, Lore Vallaeys^6^, Marije Meuwissen^7,8^, Katrien Janssens^7,8^, Patrick Verloo^9^, Benson Ogunjimi^10,11^, Dimitri Hemelsoet^12^, Program for Undiagnosed Rare Diseases (UD-PrOZA)^†^, Levi Hoste^1,2^, Lisa Roels^1,2^, Marieke De Bruyne^13,14^, Elfride De Baere^13,14^, Jo Van Dorpe^15^, Amélie Dendooven^15,16^, Anne Sieben^12,16^, Gillian I. Rice^17^, Tessa Kerre^18^, Rudi Beyaert^4,19^, Carolina Uggenti^20^, Yanick J. Crow^20,21^, Simon J. Tavernier^2,4,13,19#^, Jonathan Maelfait^3,4#^, Filomeen Haerynck^1,2#*^**

*** Correspondence:**Filomeen Haerynck, M.D., Ph.D.

Department of Pediatric Pulmonology, Infectious Diseases and Immunology, Ghent University

Hospital, Corneel Heymanslaan 10, Ghent, Belgium

[filomeen.haerynck@uzgent.be](mailto:filomeen.haerynck@uzgent.be)

Supplementary data

**S1. Case Descriptions**

**Patient 1.** Normal pregnancy and delivery at 39 weeks gestation weighing 2.9 kg with an Apgar score of 9/9. Clinical examination at birth revealed a hypospadias. A normal perinatal period was followed by some feeding difficulties and irritability in the first months of life. Positional plagiocephaly was noticed at the age of 2 months. At the age of 5 months clinical neurological examination revealed a moderate plagiocephaly, severe axial hypotonia with very limited head control, increased tone and hyperreflexia in all four limbs and bilateral abnormal plantar reflexes. CSF analysis revealed no abnormalities in white blood cells, protein or glucose levels. Fundoscopic evaluation and EEG (electroencephalogram) were both normal. Cranial ultrasound revealed a striking lenticulostriatal vasculopathy. Intracranial calcifications were seen on CT and some delayed myelination on MRI. CMV IgM and IgG were both negative in the child and mother. Screening for metabolic disorders showed normal levels of plasma lactate, ammonium and sialotransferrin, discrete lower levels of free plasma carnitine and mildly elevated levels of alanine. Urinary analysis was normal for aminoacids, organic acids, purines and pyrimidines. Laboratory analyses were also normal for thyroid, liver and kidney function test. Family history revealed an older brother with autism spectrum disorder but no abnormalities on clinical neurological examination. A follow-up at 15 months of age showed more social interaction (smiles, recognition) but delayed motor progression. Persistent abnormalities on clinical neurological examination were seen and spontaneous clonus at the ankles developed. At 16 months of age a sudden loss of consciousness occurred with a tonic gaze deviation and no reactivity to physical stimuli for about twenty minutes, suggestive for a postictal state after an epileptic seizure; treatment with valproic acid was initiated. Neuroimaging with CT and MRI revealed an old lenticulostriate ischemic lacunar cerebral infarct, not correlated with the clinical episode of epilepsy.

**Patient 2.** Normal pregnancy and caesarean delivery at 41 weeks, due to fetal heart rate decelerations, weighing 3.5 kg. The mother suffered from Klippel-Trénaunay-Weber syndrome with cutaneous capillary malformations on the right arm and both legs, but no functional disabilities. Normal perinatal period apart from a persistent bronchial hyperreactivity treated with inhalation therapy and antibiotics. She was referred to the pediatric neurology department at 1 year of age because of delayed motor progression. Clinical neurological examination revealed axial hypotonia, hypertonic quadriplegia (more prominent proximally than distally) and abnormal plantar reflexes. Neuroimaging demonstrated intracranial calcifications on CT and periventricular white matter disease, mostly in the frontal lobes, with delayed myelination seen on MRI. Cardiac and abdominal ultrasound showed no abnormalities and there were no signs of vasculopathy on cranial ultrasound. PCR assay for CMV on newborn screening card was negative. Screening for metabolic disorders demonstrated normal levels of plasma lactate, ammonium, acylcarnitines, aminoacids and sialotransferrin. CSF analysis revealed no abnormalities in aminoacid, glucose, lactate or protein concentration. No abnormal presence of red or white blood cells in the CSF. Laboratory analyses revealed no signs of anemia and normal thyroid and kidney function, but mildly elevated liver enzymes (AST, ALT). Evaluation at the age of 3 years revealed persistent clinical neurological abnormalities, but progression of her cognitive and motoric capabilities. MRI was repeated and showed only mild signal abnormalities in the periventricular white matter.

**Patient 3.** Normal pregnancy and delivery at 40.5 weeks gestation weighing 4.0 kg with an Apgar score of 9/9. He was the second child of healthy, non-consanguineous parents. Family history was normal. By the age of 2 months irritability and feeding difficulties were obvious. At 6 months of age, he was clinically evaluated due to developmental delay characterized by absent social interaction and motor delay with spasticity and axial hypotonia. Fundoscopic evaluation and EEG were both normal. CT imaging of the brain revealed bilateral calcifications located periventricular and in the corpora of the lateral ventricles and basal ganglia. MRI showed mild atrophy and pronounced periventricular leukodystrophy. Infectious screening for TORCH was negative. Cardiac ultrasound was normal. CSF analysis showed high levels of albumin (170 mg/dL) and elevated immunoglobulin M (<0.4 mg/dL; 0-0.13). Metabolic screening on CSF revealed normal pyruvate, aminoacids and glucose. White blood cells in CSF were normal except for some monocytes with prominent cytosolic vacuolization. Galactocerebrosidase activity was normal. Clinical examination confirmed axial hypotonia with hypertonia in all four limbs with hyperreflexia. Bilateral Rossolimo reflex and Babinski reflex were present at 18 months. Routine laboratory analysis of liver enzymes and kidney function showed no abnormalities. EMG was performed and revealed normal sensory and motor conduction. Head circumference evolution fell from P90 to P10-50. A follow-up MRI at 18 months showed periventricular leukodystrophy. Reevaluation of the CSF showed increased levels of neopterins (51 nmol/L, reference value 9-30) and biopterins (144 nmol/L, reference value 10-30) without elevation of IFN-α. He was in follow-up at the endocrinology department because of bilateral cryptorchidism. At 6 years of age he suffered from a swollen knee joint and developed clinical synovitis of MCP2-3 in the left hand. In addition, he developed a chronic conjunctivitis and suffered from plantar erythema. Serology for autoimmune rheumatic disease including ANF, RF, anti-CCP, and ANCA was normal. Neurological follow-up revealed persistent abnormalities in cognitive and motor developmental without further regression. At 18 years of age, he developed a neurogenic bladder and suffered from recurrent urinary tract infections and treatment with intermittent catheterization was started. In addition, he complained of regurgitation and vomiting. At age 19 years he was admitted to hospital because of a bilateral tonic-clonic seizure. Laboratory analysis revealed signs of mild hemolytic anemia (presence of schistocytes and decreased haptoglobin) and unexplained renal insufficiency (creatinine of 2.85 mg/dl, eGFR 30 ml/min) with arterial hypertension. Fundoscopic examination was normal. Cardiac workup showed elevated troponins without signs of ischemia on ECG. An additional cardiac ultrasound revealed normal functional and structural evaluation, but a pericardial effusion was noticed (5-10mm) without hemodynamic compromise. There was no improvement in renal function despite adequate rehydration and exclusion of a postrenal obstruction. Renal imaging showed a profound reduction of corticomedullary differentiation. A kidney biopsy was not performed. The patient died one month later following palliative home care. Postmortem examination of the brain revealed mild cerebellar atrophy and leukodystrophy. Microscopic evaluation revealed spongiosis in the substantia nigra and nucleus dentatus. Microcalcifications are present in deep white matter (most prominent in thalamus, basal ganglia, and cerebellum) and arterial walls. Several regions of demyelination with a significant increase of macrophages and clusters of activated microglial cells located in the deep white matter or perivascular. Histopathology of the kidney showed hallmarks of severe acute and chronic thrombotic microangiopathy. HE staining showed glomeruli with fibrin thrombi and arterioles with intramural fibrin precipitation. Silver staining (Jones methenamine) showed glomeruli with mesangiolysis, endothelial swelling, and segmental sclerosis as well as ‘onion-skinning’ of arterioles. Immunofluorescence staining for immune complexes and complement deposition (IgG, IgA, IgM, C1q, C3) was negative, excluding an immune-complex pathogenesis.

**AGS patient controls.** A boy harboring compound heterozygous *RNASEH2B* mutations (c.529G>A and c.254T>C) presented with developmental delay and neurologic regression. A brain MRI revealed severe leukodystrophy and cortical atrophy. He is currently 12 years with severe hypotonia on the last clinical visit. A 3-year-old girl carrying homozygous pathogenic *TREX1* mutations presented with hypotonia, nystagmus, and epilepsy. Cranial ultrasound revealed bilateral lenticulostriatal vasculopathy and leukodystrophy on brain MRI. She is currently 13 years and has severe cognitive and motoric impairment. Genetic analysis in a girl presenting with hypertonia, intracranial calcifications and leukodystrophy demonstrated compound heterozygous mutations in *SAMHD1* (p.E37X and exon 5 deletion). She is currently 12 years and recently treatment was started with a Janus kinase (JAK) inhibitor (Tofacitinib) because of persistent chilblains with a beneficial response.

**S2. 20x coverage by WES of AGS associated genes**

|  | **P1** | **P2** | **P3** |
| --- | --- | --- | --- |
| TREX1 | 100% | 100% | 100% |
| RNASEH2A | 100% | 100% | 100% |
| RNASEH2B | 100% | 61.1% | 92.9% |
| RNASEH2C | 100% | 100% | 100% |
| SAMHD1 | 100% | 79% | 98.5% |
| ADAR1 | 100% | 99.5% | 100% |
| IFIH1 | 100% | 81.2% | 95.3% |
| LSM11 | 97.3% | 98.2% | 100% |
| RNU7-1 | NA | NA | NA |

**S3. Secondary structure of other reported RNU7-1 mutations**

**
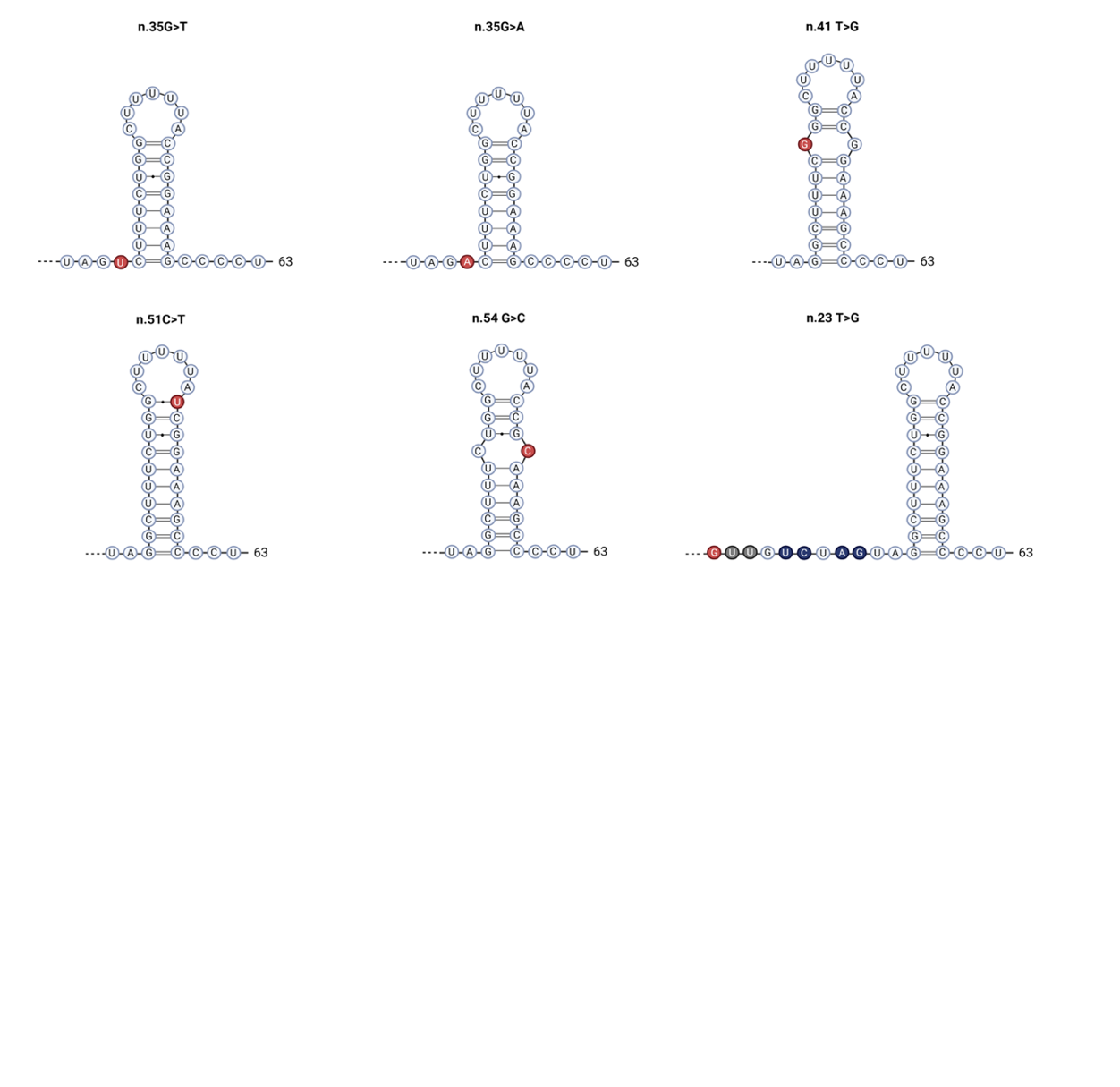
**

**Fig. S3.** Secondary structure of reported pathogenic *RNU7-1* stem-loop mutations associated with AGS.

**S4. Expression of LSM11 in primary patient fibroblasts**

**
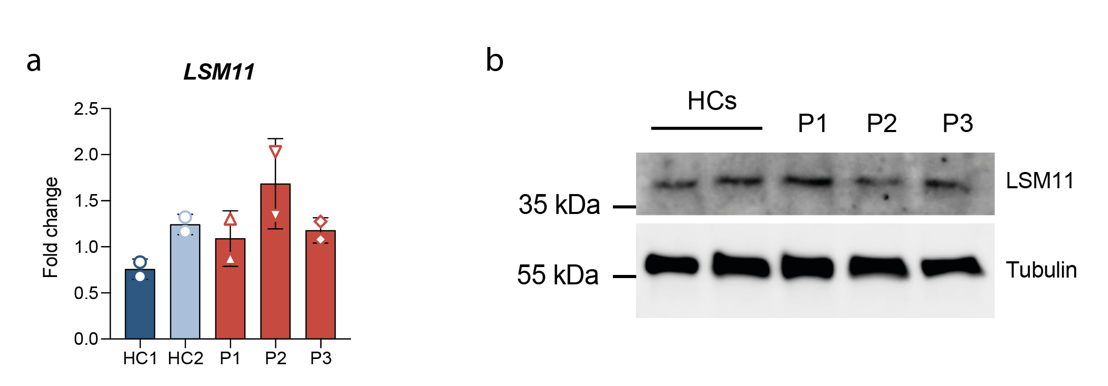
**

**Fig. S4.** (**a**) mRNA expression of *LSM11* in fibroblasts of AGS patients (P1, P2, P3) compared to HCs (n=2), presented as fold change normalized to cellular *SDHA* relative to the values for HCs. (**b**) Endogenous LSM protein abundance in fibroblasts from HCs (n=2) and AGS patients (P1, P2, P3), assessed by immunoblotting (IB) with anti-LSM11. Tubulin served as loading control.

**S5. ISG score in whole blood**

|  | **P1** | **P2** | ***SAMHD1*** | ***RNASEH2B*** | ***TREX1*** |
| --- | --- | --- | --- | --- | --- |
| IFI27 | 19.54 | 9.15 | 76.5 | 785.75 | 73.73 |
| IFI44L | 10.02 | 12.6 | 34.98 | 56.52 | 45.99 |
| IFIT1 | 5.26 | 10.26 | 10.53 | 11 | 17.31 |
| ISG15 | 4.22 | 6.34 | 11.9 | 13.38 | 12.39 |
| RSAD2 | 10.08 | 11.36 | 15.29 | 21.5 | 34.9 |
| SIGLEC1 | 2.34 | 3.87 | 10.29 | 32.33 | 27.3 |
| CMPK2 | 2.59 | 2.99 | 4.71 | 9.06 | 8.14 |
| DDX60 | 3.15 | 3.7 | 5.47 | 7.02 | 6.53 |
| EPSTI1 | 7.18 | 6.2 | 10.42 | 10.92 | 12.78 |
| FBXO39 | 3.78 | 4.11 | 3.37 | 6.89 | 5.76 |
| HERC5 | 2.07 | 4 | 3.86 | 4.77 | 7.99 |
| HES4 | 1.85 | 2.1 | 1.6 | 6 | 3.52 |
| IFI44 | 4.48 | 6.73 | 12.32 | 19.19 | 18.98 |
| IFI6 | 5.32 | 6.1 | 11.31 | 12.6 | 14.71 |
| IFIH1 | 1.62 | 2.02 | 2.48 | 2.62 | 2.46 |
| IRF7 | 1.46 | 1.76 | 3.1 | 3.54 | 3.92 |
| LAMP3 | 3.93 | 3.17 | 7.71 | 9.35 | 5.43 |
| LY6E | 4.54 | 3.8 | 5.84 | 7.54 | 10.81 |
| MX1 | 3.12 | 3.99 | 8.17 | 7.61 | 13.04 |
| NRIR | 2.66 | 3.88 | 6.78 | 20.75 | 8.38 |
| OAS1 | 5.8 | 7.32 | 11.6 | 11.75 | 16.83 |
| OASL | 3.08 | 3.47 | 3.73 | 3.93 | 5.45 |
| OTOF | 16.71 | 2.76 | 13.2 | 52.81 | 35.42 |
| SPATS2L | 2.79 | 2.29 | 4.31 | 8.04 | 7.39 |
| **ISG score*** | **3.855** | **3.935** | **7.94** | **10.135** | **11.6** |

**Table S5.** Median fold induction of 24 ISG transcripts and ISG score in patients with mutations in *RNU7-1* (n=2), *SAMHD1* (n=1), *RNASEH2B* (n=1), or *TREX1* (n=1) mutations compared to healthy controls (n=27). *Positive ISG score ≥2.758.

**S6. Expression of STAT1 and STAT2**

**
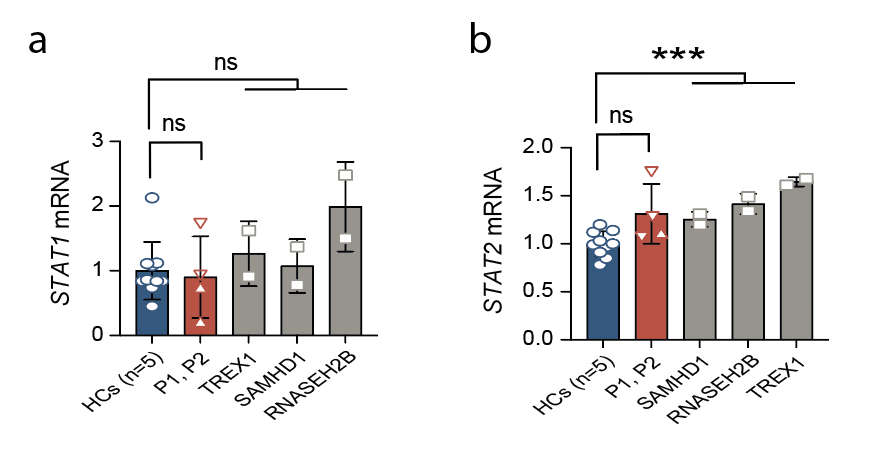
**

**Fig. S6.** mRNA expression of *STAT1* (**a**) and *STAT2* (**b**) in whole blood of *RNU7-1* mutated patients and AGS patients harboring biallelic mutations in *SAMHD1* (n=1), *RNASEH2B* (n=1) or *TREX1* (n=1) compared to HCs (n=5), presented as fold change normalized to cellular *SDHA* relative to the values for HCs. Data shown are representative for two independent experiments (mean and s.d. of n = 2 technical replicates). ***P < 0.001, by non-parametric unpaired Mann-Whitney U test.

**S7. Mortality rate of AGS 1-8 genotypes**

**a**

**
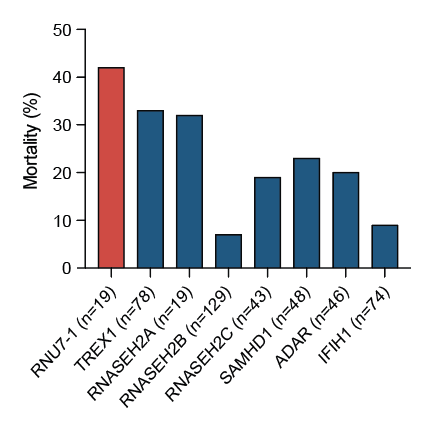
**

**b**

| ***RNU7-1* patients** | **Cause of death** | **Age (years)** |
| --- | --- | --- |
| **1** | Epileptic seizure | 11 |
| **2** | Liver failure | 7 |
| **3** | Liver failure | 9 |
| **4** | Renal failure | 7 |
| **5** | Renal failure | 9 |
| **6** | Unknown | 8 |
| **7** | Liver failure/ Renal failure | 9 |
| **8** | Epilepsy / Renal failure | 19 |

**Fig. S7.** (**a**) Mortality rate calculated from reported AGS genotypes including *RNU7-1* (n=19) (this publication and [1], TREX1 (n=78), RNASEH2 (n=19), RNASEH2B (n=129), RNASEH2C (n=43) [2], ADAR (n=46) [3], IFIH1 (n=74) [4]. (**b**) Cause and age of death in AGS patients with *RNU7-1* mutations.

**S8. Primer sequences**

|  | **Forward (5’ to 3’)** | **Reverse (5’ to 3’)** |
| --- | --- | --- |
| **HIST1H1C** | ACACCGAAGAAAGCGAAGAA | GCTTGACAACCTTGGGCTTA |
| **HIST1H2AC** | GACCATTGCTCAGGGCGGCGTCCT | CCTTCTACCTACAAGCAGTGAGGTT |
| **H1FX** | GTGGTTCGACCAGCAGAATG | GAGCTTGAAGGAACCGTTGG |
| **H3F3A** | AAAGCCGCTCGCAAGAGTGCG | ACTTGCCTCCTGCAAAGCAC |

**References**

1. Uggenti C, Lepelley A, Depp M et al. cGAS-mediated induction of type I interferon due to inborn errors of histone pre-mRNA processing. Nat Genet. 2020;52:1364-1372.

2. Crow YJ, Chase DS, Lowenstein Schmidt J et al. Characterization of human disease phenotypes associated with mutations in TREX1, RNASEH2A, RNASEH2B, RNASEH2C, SAMHD1, ADAR, and IFIH1. Am J Med Genet A. 2015;167A:296-312.

3. Rice GI, Kitabayashi N, Barth M et al. Genetic, Phenotypic, and Interferon Biomarker Status in ADAR1-Related Neurological Disease. Neuropediatrics. 2017;48:166-184.

4. Rice GI, Park S, Gavazzi F et al. Genetic and phenotypic spectrum associated with IFIH1 gain-of-function. Hum Mutat. 2020;41:837-849.
